# An insight in magnetic field enhanced zero-valent iron/H_2_O_2_ Fenton-like systems: Critical role and evolution of the pristine iron oxides layer

**DOI:** 10.1038/srep24094

**Published:** 2016-04-07

**Authors:** Wei Xiang, Beiping Zhang, Tao Zhou, Xiaohui Wu, Juan Mao

**Affiliations:** 1 School of Environmental Science and Engineering, Huazhong University of Science and Technology, Wuhan, 430074, P. R. China.; 2 Key Laboratory of Water and Wastewater Treatment (HUST), MOHURD, Wuhan, 430074, P. R. China.

## Abstract

This study demonstrated the synergistic degradation of 4-chlorophenol (4-CP) achieved in a magnetic field (MF) enhanced zero-valent iron (ZVI)/H_2_O_2_ Fenton-like (FL) system and revealed an interesting correlative dependence relationship between MF and the pristine iron oxides layer (Fe_x_O_y_) on ZVI particles. First, a comparative investigation between the FL and MF-FL systems was conducted under different experimental conditions. The MF-FL system could suppress the duration of initial lag degradation phase one order of magnitude in addition of the significant enhancement in overall 4-CP degradation. Monitoring of intermediates/products indicated that MF would just accelerate the Fenton reactions to produce hydroxyl radical more rapidly. Evolutions of simultaneously released dissolved iron species suggested that MF would not only improve mass-transfer of the initial heterogeneous reactions, but also modify the pristine ZVI surface. Characterizations of the specific prepared ZVI samples evidenced that MF would induce a special evolution mechanism of the ZVI particles surface depending on the existence of Fe_x_O_y_ layer. It comprised of an initial rapid point dissolution of Fe_x_O_y_ and a following pitting corrosion of the exposed Fe^0^ reactive sites, finally leading to appearance of a particular rugged surface topography with numerous adjacent Fe^0^ pits and Fe_x_O_y_ tubercles.

In the past decades, zero valent iron (ZVI) technologies have been proved as effective approaches to remove various aqueous organic/inorganic contaminants^1^. As a common and cost-effective transition metal, ZVI can act as not only reducing agent directly but also electron-donor in attending oxidative reactions^2^,^3^,^4^,^5^,^6^,^7^,^8^. It is well established that ZVI can be applied in ground water remediation for direct reduction of heavy metal ions e.g. arsenate and/or reductive dehalogenation of organic compounds e.g. trichloroethylene^3^,^4^,^5^. Besides, ZVI-inducing advanced oxidation processes (AOPs) have become more attractive since they are capable of completely decomposing and mineralizing harmful organic contaminants^6^,^7^,^9^,^10^. It has been reported that ZVI could efficiently catalyze common oxidants such as H_2_O_2_, O_2_, and persulfate to generate more reactive radicals, e.g. hydroxyl radicals (OH•) and sulfate radicals^6^,^8^,^11^,^12^,^13^,^14^,^15^. These radicals are of high redox potential and able to oxidize numerous recalcitrant organic pollutants non-selectively^6^,^7^,^8^,^11^.

As a classic AOP involving the reaction of Fe^2+^ and H_2_O_2_ to generate OH•, Fenton reaction is expected to be applied in wastewater treatment due to its high treatment efficiency and mild operational conditions^11^. However, direct use of ferrous salt catalyst (FeSO_4_) will result in limitations such as rapid and useless consumption of Fe^2+^ as well as overload of ferric ions in effluents^12^,^14^. ZVI is an appropriate alternative Fenton catalyst, taking advantage of its continuous supply of Fe^2+^ during iron corrosionandpromotion inrecycling of ferric iron at the iron surface^6^,^13^,^15^. It was demonstrated that the ZVI/H_2_O_2_ system, generally called heterogeneous Fenton-like (FL) system, could effectively degrade many recalcitrant organic pollutants^12^,^13^,^16^.

Undoubtedly, the efficiency of ZVI technologies depends strongly on characteristics of the ZVI materials used. Commercial iron powders are generally adopted in the ZVI/H_2_O_2_ systems^6^,^12^,^15^,^17^. However, undesirable phenomena of initial lag reaction periods were observed, mainly attributing to existence of passive oxides films around the ZVI particles^12^,^18^,^19^. Occurrence of outer oxides filmscan be observed during the manufacture and storage procedures of commercial iron powders^1^,^17^,^18^,^19^. It makes the ZVI particles atmospherically stable and block the electron transfer from inner Fe^0^ to the surface^1^,^20^. There are several methods to improve the surface reactivity of commercial ZVI materials, e.g. acid washing^5^,^21^, H_2_-reduction^22^,^23^, sonication^24^, and electrochemical reduction^25^. Nevertheless, disadvantages including operational complexity, additional cost, and/or chemical wastes should be considered for practical applications of these pretreatment methods^1^.

Recently, weak magnetic field (MF, <70 mT) was regarded as a promising cost-effective and environmental friendly tool to benefit the ZVI technologies^1^. It was reported that WMF not only significantly accelerated the removal of heavy metal ions (Se(IV), As(III)/As(V) and Cu(II)) by ZVI over a wide pH range^18^,^19^,^26^,^27^,^28^,^29^,^30^, but also improved the degradation of organic pollutants in Fe^0^/O_2_ or Fe^0^/persulfate systems^29^,^30^. Based on a series of studies, Guan’s group concluded an enhancement role of weak MF in neutral ZVI systems^1^. On one hand, MF can enhance the mass transport by the generated Lorentz force (F_L_) which would give rise to convection in the solution^1^. Further, MF stimulates the breakdown of the passive film and eventually localized corrosion on the surface of ZVI particles^31^. It was due to the fact that the induced gradient magnetic force (GMF) could move paramagnetic Fe^2+^ along the higher field gradient at the surface of ZVI particle, thus causing localized galvanic couples on the particle surface^31^.

To our best knowledge, the introduction of weak MF into acidic ZVI/H_2_O_2_ Fenton like system has been never investigated. In a preliminary experiment, we have found sharp synergistic degradation of 4-chlorophenol (4-CP) achieved in a ZVI/H_2_O_2_ system (pH = 3) with MF irradiation. It was assumed that MF would probably accelerate the depassivation of commercial atmospherically stable ZVI powders (AS-ZVI) and thus increase its catalyzing reactivity. Herein, we want to further reveal the enhancement mechanism of MF on the 4-CP degradation in such acidic Fenton like systems. The contents include (i) comparative degradation of 4-CP in the ZVI/H_2_O_2_ system (FL) and the MF-assisted ZVI/H_2_O_2_ system (MF-FL) under different experimental conditions, (ii) proposed degradation pathways based on the examinations of intermediates/products in the FL and MF-FL systems, and (iii) exploration of surface characteristics of different ZVI samples made for specific purposes. Finally, a special interactive role between MF and the pristine iron oxides layer could be revealed to explain the synergistic 4-CP degradation achieved in the MF-FL system.

## Results and Discussion

### Comparative 4-CP degradation in the ZVI/H_2_O_2_ systems with/without MF

It was found that 4-CP was recalcitrant to the treatment of ZVI or H_2_O_2_ alone, with MF present or not. Introduction of MF brought obvious enhancement on the 4-CP degradation in the ZVI/H_2_O_2_ Fenton-like system. To explore the effect of MF, comparative 4-CP degradation in the FL system and its corresponding system with MF (MF-FL system) was investigated. According to the obtained data (Supplementary Figs S1–S4) and literature^12^, the degradation procedure of 4-CP could be basically divided into an initial lag degradation phase (phase I) and a following rapid degradation phase (phase II). The pseudo-first-order kinetics could be used to apply in both phases. An example of the two-phase identification is given in Supplementary Fig. S5. Significant analyses for the related obtaining data have been also conducted (Supplementary Tables S1 and S2).

### Effect of initial ZVI dosage on the 4-CP degradation in the two comparative systems

**Figure 1:**
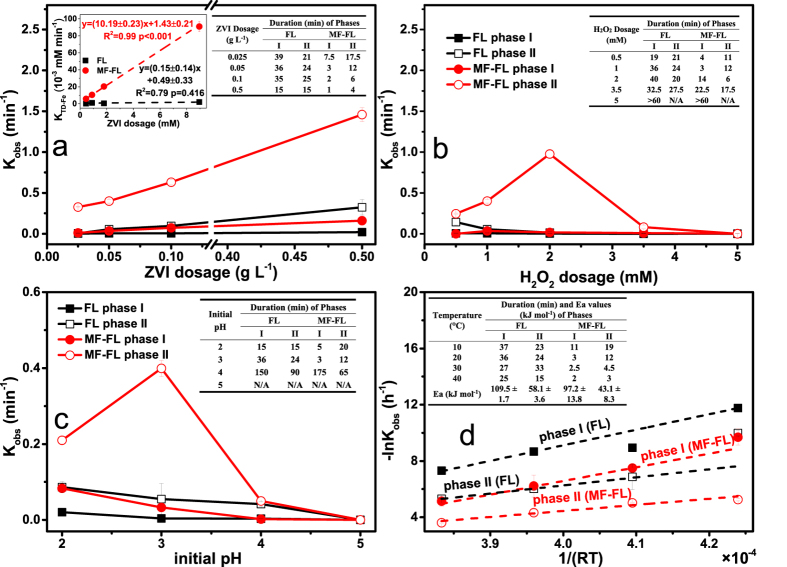
Comparative 4-CP degradation of in the FL and MF-FL systems with the variation of (**a**) ZVI dosage, (**b**) H_2_O_2_ dosage, (**c**) initial pH, and (**d**) reaction temperature. (Except the investigated parameter, conditions were: initial pH of 3, 0.05 g L^−1^ ZVI, 25 mg L^−1^ 4-CP, 1.0 mM H_2_O_2_ and 20 °C). Inset figure in (**a**) illustrates the linear-correlation of the accumulation rate of total dissolved iron and the ZVI dosage. The error bars represent the standard deviation based on triplicate experiments. Inset tables in (**a**–**d**) present the two-phase durations in the FL and MF-FL systems, with different investigated parameters, respectively. The related Ea values are also included in the inset table of (**d**). The uncertainties of Ea values were determined from the standard error of the linear regression.

Figure 1a presents the 4-CP degradation in the two comparative systems with the ZVI dosage varying from 0.025 to 0.5 g L^−1^. Increasing ZVI dosage obviously increased the 4-CP degradation rate constants (k_obs_(4-CP)) for both systems. As compared to the FL system, the MF-FL system not only leaded to remarkable improvements in the 4-CP degradation in both phase I and phase II, but also dramatically shorten the duration of phase I from tens to several minutes. Simultaneous release of the dissolved iron species was examined (Supplementary Fig. S1). The corresponding release of Fe^2+^ correlated positively with the 4-CP degradation. It indicated that MF could accelerate the release of dissolved iron species and thus improve the homogenous Fenton reactions^29^. In both the FL and MF-FL systems, the release behavior of total dissolved iron species could be fitted by the zero-order kinetic. As the ZVI dosage increased, the release rate of total dissolved iron (k_obs_(TD-Fe)) increased linearly in both systems (inset figure in Fig. 1a). Moreover, value of the ZVI-specified k_obs_(TD-Fe) in the MF-FL system was c.a. 56 times higher than that in the FL system. Apparently, increasing ZVI amounts was beneficial to the MF-enhancing release of dissolved iron species. A similar observation was also reported wherein weak MF could enhance the release of Fe^3+^ significantly in a ZVI/persulfate system^29^. Commonly, pristine ZVI particles are incrusted by formed iron oxides (Fe_x_O_y_) layer during their production and storage^1^,^20^. Existence of the Fe_x_O_y_ layer will result in initial lag reaction phases^18^,^32^ and weak MF can be used to stimulate breakdown of the passive films and shorten the initial lag phases^18^,^32^. As a result, a positive relationship between MF and the pristine ZVI particles could be expected in this study.

### Effect of initial H_2_O_2_ dosage

From Fig. 1b, it can be seen that the initial H_2_O_2_ dosage would exhibit different effects on the 4-CP degradation between the FL and MF-FL systems. In the FL system, the 4-CP degradation was inhibited with the increase of initial H_2_O_2_ dosage. The related duration of phase I was also extended. With a much higher H_2_O_2_ dosage of 5.0 mM, the 4-CP degradation was almost inhibited, even if the reaction time prolonged to 60 min. It was because that excessive amounts of H_2_O_2_ would inactivate the ZVI surface and inhibit the release of ferrous ion^33^,^34^. Fe^2+^ was found to increase gradually in the case of 0.5 mM H_2_O_2_ whereas it almost not be detected in the cases of high H_2_O_2_ dosage of 5.0 mM (Supplementary Fig. S2). As compared to the FL system, an overall improvement in the 4-CP degradation was observed in the MF-FL system, at the H_2_O_2_ dosage range of from 0.5 to 2 mM. Moreover, as the dosage increased in this range, the k_obs_(4-CP)_phase I_ decreased gradually but the k_obs_(4-CP)_phase II_ increased obviously. Although a maximum k_obs_(4-CP)_phase II_ value was obtained in the case of 2.0 mM H_2_O_2_, duration of the related phase I was raised one order of magnitude from 3–4 to 14 min. The 4-CP degradation patterns under different H_2_O_2_ dosages were also in accordance with their corresponding release of dissolved iron species (Supplementary Fig. S2). In addition, the 4-CP degradation in the MF-FL system was almost inhibited with 5.0 mM H_2_O_2_, and negligible aqueous iron was detected. Therefore, the enhancement of 4-CP degradation in the MF-FL system could be mainly attributed to the effective release of dissolved Fe^2+^ in the presence of relatively concentrated H_2_O_2_. MF could activate ZVI particles to generate available fresh Fe^0^ sites due to a “pitting corrosion” effect^18^, thus leading to more efficient effective Fenton reactions^22^,^35^.

### Effect of initial pH

As shown in Fig. 1c, the 4-CP degradation in both systems occurred in the initial pH range of 2–4, whereas it was almost suppressed with an initial pH of 5. Acidic conditions favored the 4-CP degradation in the FL system and the best performance was obtained in the case of initial pH 2. It would ascribe to the rapid release of dissolved Fe^2+^ and Fe^3+^ as a result of fast proton(H^+^)-dissolution of the Fe_x_O_y_ layer on the ZVI surface (Supplementary Fig. S3)^18^,^36^. Meanwhile, the MF-FL system leaded to good enhancements in the 4-CP degradation at initial pH range of 2–3 but marginal effect at initial pH 4 (Fig. 1c). Similar to the traditional homogeneous Fenton system^37^, the MF-FL system also presented the optimal 4-CP degradation efficiency at pH of 3, probably due to that more amounts of Fe^2+^ were released in the case of pH 3 than that in the case of pH 2 (Supplementary Fig. S3). Higher concentrations of Fe^3+^ than Fe^2+^ was found in the case of pH 2, since the net oxidation of Fe(II) with the generated radicals species would occur at lower pH^38^. At initial pH of 4, the simultaneous releases of dissolved iron species were marginal in either the FL or MF-FL system, until the 4-CP was decomposed completely at a prolonged reaction time of 240 min (Supplementary Fig. S3). It indicated that heterogeneous Fenton-like reactions controlled by surface-bonded Fe(II) would be dominant^7^. As the 4-CP decomposed, low molecule organic acids would be formed^12^,^29^, leading to gradual pH decrease down to about 3.5 at 240 min. Thereafter, a sudden release of dissolved iron species happened in the MF-FL system. However, neither 4-CP degradation nor release of dissolved iron species was observed in the case of pH 5 during the whole reaction time of 1500 min. It indicated that the effective proton-dissolution of the Fe_x_O_y_ layer upon the ZVI surface would be essential to the release of dissolved iron species in the MF-FL system. Therefore, the main synergistic role of MF in the system was supposed to its enhancement in the surface dissolution and corrosion of ZVI^39^, rather than the heterogeneous 4-CP degradation reactions controlled by surface-attaching Fe(II).

### Effect of reaction temperature

Effect of the reaction temperatures ranging from 10 to 40 °C on the 4-CP degradation was also evaluated. It was found that the relationship between the reaction temperature (K) and k_obs_(4-CP)_phase I_ or k_obs_(4-CP)_phase II_ could be applied by the Arrhenius equation^40^, in both systems. The related linear-fitting curves as well as the Arrhenius activation energy (E_a_) values are shown in Fig. 1d. With related to the FL system and the MF-FL system, the value of E_a_(phase I) was 110.3 ± 1.7 and 97.2 ± 13.8 kJ mol^−1^ while the value of E_a_(phase II) was 57.2 ± 3.6 and 43.1 ± 8.3 kJ mol^−1^, respectively. The related statistical test of the Ea values was presented in Supplementary Table S3. In both systems, E_a_ of the phase I was approximately double that of the phase II (P = 0.016). It indicated that the reactions in the phase I were mainly controlled by the interfacial mass transfer^4^, whereas the reactions in the phase II were limited by chemical reaction rate^41^. As compared to the FL system, the MF-FL system obtained relatively lower E_a_(phase I) and E_a_(phase II). Although the Ea values were not significant different between the two systems (P = 0.063), the duration of phase I was reduced about one of order magnitude in the MF-FL system (inset table in Fig. 1d). It suggested that MF would not only improve mass transfer of the initial surface-bond reactions, but also lead to simultaneous variation on the surface properties of the pristine ZVI^19^,^39^.

### Evolutions of 4-CP degradation intermediates/products and proposed degradation pathways in the two comparative systems

**Figure 2:**
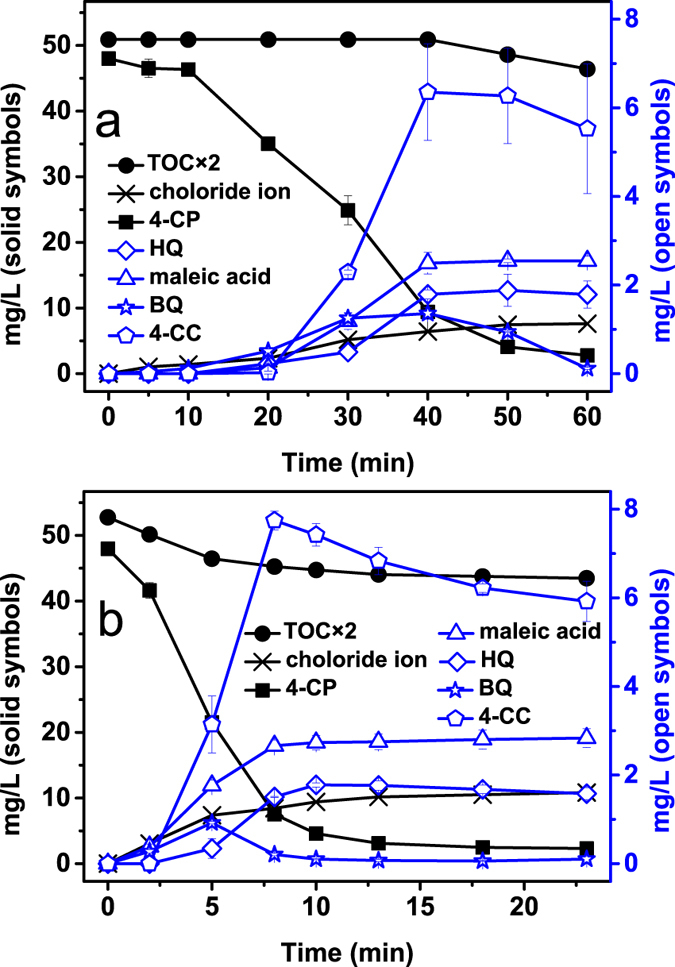
Time-dependent evolutions of TOC, chloride ion 4-CP and its degradation intermediates, HQ, maleic acid, BQ and 4-CC, in (**a**) the FL system, and (**b**) the MF-FL system. (Conditions: initial pH of 3, 0.1 g L^−1^ ZVI, 50 mg L^−1^ 4-CP, 1.0 mM H_2_O_2_ and 20 °C). The error bars represent the standard deviation based on duplicate experiments.

**Figure 3:**
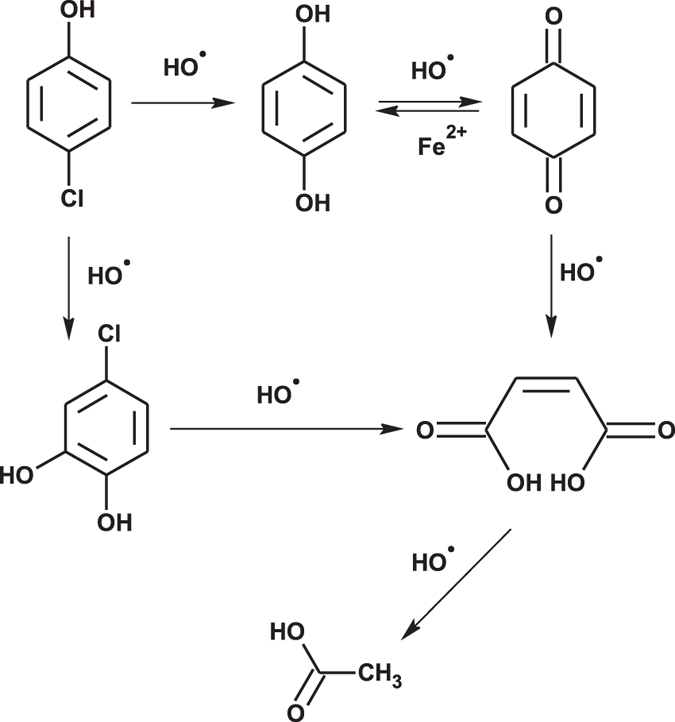
An illustration of the proposed 4-CP degradation pathways in the FL and MF-FL systems.

It was demonstrated that OH• was the dominant oxidant in both the FL system and MF-FL system, through the methanol quenching experiment and the spin trapping examinations (Supplementary Fig. S6). Examinations of the 4-CP degradation intermediates/products in both systems were conducted by HPLC-ESI-MS, GC-MS, HPLC and IC, respectively. Chloride ion as well as four main organic intermediates, i.e. hydroquinone (HQ), benzoquinone (BQ), 4-chlorocatechol (4-CC) and maleic acid, were identified (Supplementary Fig. S7). Figure 2 shows the evolutions of the five intermediates/products with the elapse of reaction time. Similar evolution trends were observed in the FL and MF-FL systems, while formation and disappearance of the five intermediates/products did occur earlier in the latter system. It indicated that MF would accelerate the Fenton reactions to produce OH• more rapidly, in lieu of vary the 4-CP degradation reactions per se. According to the results and literatures^12^,^42^,^43^,^44^,^45^, a mutual scheme of 4-CP degradation pathways in the two systems could be proposed as presented in Fig. 3. It comprised of two pathways under OH• attacking of different position in the aromatic ring. One was the direct dechlorination of 4-CP molecule, leading to the formation of HQ and BQ. The other was electrophilic addition of OH• at *ortho* position of OH group on the 4-CP molecule, resulting in the formation of 4-CC as well as its derivates catechol and 1,2,4-benzenetriol^12^. Further OH• mineralization would lead to the ring cleavage of the aromatic intermediates and formation of maleic acid, acetic acid, formic acid, CO_2_, H_2_O and Cl^−^12,42.

### Comparative 4-CP degradation in the absence/presence of the Fe_x_O_y_ layer

**Figure 4:**
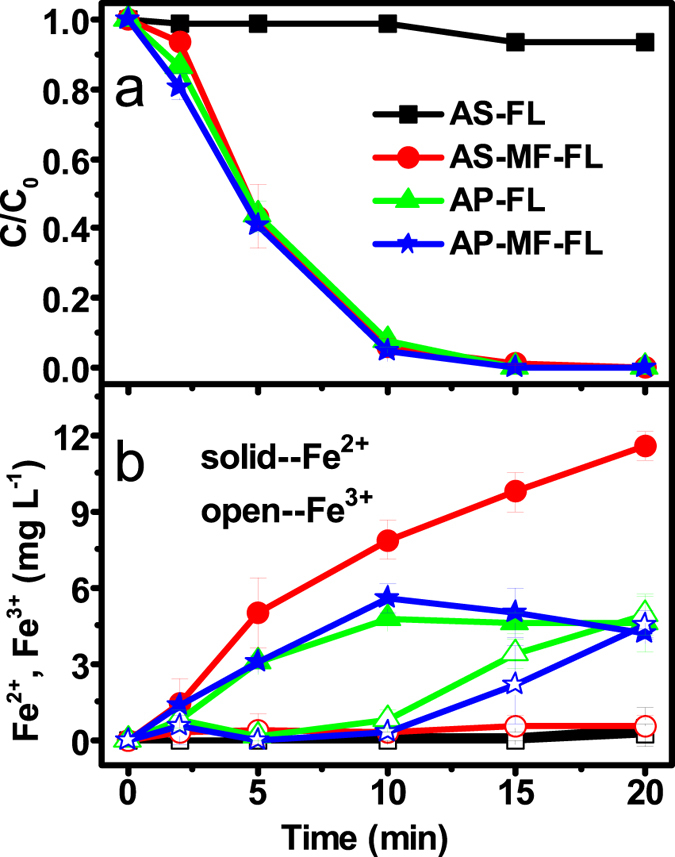
Comparative (**a**) 4-CP degradation, and (**b**) corresponding evolution of Fe^2+^/Fe^3+^ in the FL system and the MF-FL system, based on the AS-ZVI and the AP-ZVI, respectively. (Conditions: 25 mg L^−1^ 4-CP, 0.05 g L^−1^ ZVI, 1.0 mM H_2_O_2_, initial pH of 3, 20 °C). The error bars represent the standard deviation based on triplicate experiments.

As described above, the main promotional role of MF would be accelerating the release of Fe^2+^ due to the enhancement in depassivation and corrosion of the pristine ZVI. To further clarify it, an acid-pretreated ZVI (AP-ZVI) was prepared since acid pretreatment could effectively remove the passive Fe_x_O_y_ layer^21^. Afterwards, the degradation of 4-CP was investigated in the FL and MF-FL systems, by using the pristine ZVI (atmospherically stable, AS-ZVI) and the AP-ZVI, respectively. As shown in Fig. 4a, MF significantly enhanced the 4-CP degradation in the FL system based on AS-ZVI rather than AP-ZVI. Except the FL system based on AS-ZVI, all other three systems exhibited similarly rapid patterns for the 4-CP degradation. Corresponding time-dependent evolutions of Fe^3+^ and Fe^2+^ were observed similarly in the FL and MF-FL systems based on AP-ZVI. However, in the systems based on AS-ZVI, Fe^2+^ was released significantly more rapid under the MF radiation whereas the appearance of Fe^3+^ was negligible in both systems. Furthermore, faster accumulation of Fe^2+^ and total dissolved iron were achieved in the MF-FL system based on AS-ZVI than AP-ZVI (Fig. 4b and Supplementary Fig. S8). It evidenced that the Fe_x_O_y_ layer should be requisite in manifesting the significant synergistic effect of MF.

### Characterizations of the related ZVI samples

**Figure 5:**
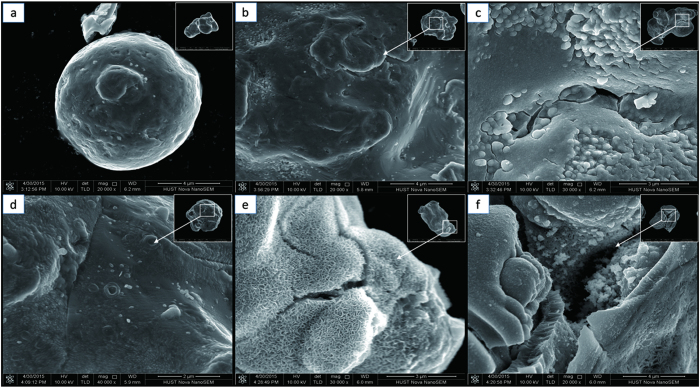
SEM images of (**a**) the pristine AS-ZVI (**b**) the reacted AS-ZVI (FL, 10 min) (**c**) the reacted AS-ZVI (MF-FL, 10 min), (**d**) the original AP-ZVI, (**e**) the reacted AP-ZVI (FL, 10 min), and (**f**) the reacted AP-ZVI (MF-FL, 10 min).

Figure 5 presents the SEM images of the related ZVI samples. It can be seen that the surface of AS-ZVI particles was relatively smooth despite the existence of individual imperfections. After 10 min treatment in the FL system, the AS-ZVI surface remained almost unchanged and only a little of new imperfections appeared. Nevertheless, the AS-ZVI treated in the MF-FL system exhibited a distinctive morphology of its surface that occupied by quantities of pits, tubercles, and even cracks (Fig. 5c). Apparently the presence of MF brought an unconventional evolution of the AS-ZVI surface. Acid-pretreatment of AS-ZVI could lead to a coarser and uneven surface (Fig. 5d). Spiculate iron oxides were observed to appear and almost cover the surface of AP-ZVI particles collected from the FL system (10 min), while clusters of iron oxides partly covered the surface of AP-ZVI particles from the MF-FL system (Fig. 5e,f). The difference was probably due to that weak MF could accelerate the transformation of amorphous iron (hdyro)oixdes to lepidocrocite^18^.

**Figure 6:**
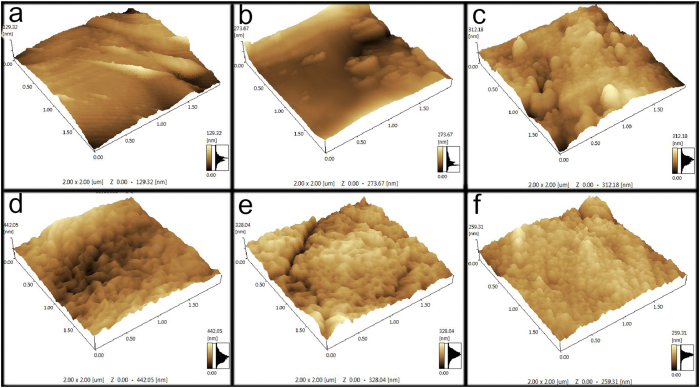
AFM images of (**a**) the pristine AS-ZVI (**b**) the reacted AS-ZVI (FL, 10 min) (**c**) the reacted AS-ZVI (MF-FL, 10 min), (**d**) the original AP-ZVI, (**e**) the reacted AP-ZVI (FL, 10 min), and (**f**) the reacted AP-ZVI (MF-FL, 10 min).

Furthermore, AFM images of the corresponding ZVI samples are shown in Fig. 6. It was found that the topographies of the pristine and FL-treated AS-ZVI particles were plain-like similarly. However, after the MF-FL treatment the AS-ZVI particles presented a particular rugged surface with numerous adjacent pits and tubercles between which the height difference was about 120 nm averagely (Supplementary Fig. S9). Besides, similar AFM topographies of the three AP-ZVI particles (unreacted, FL-treated and MF-FL treated) were observed. It could be concluded that MF would only accelerate the migration of dissolved iron species^18^, although the acid pretreatment could cause an uneven ZVI surface of Fe^0^ fresh sites^1^.

**Figure 7:**
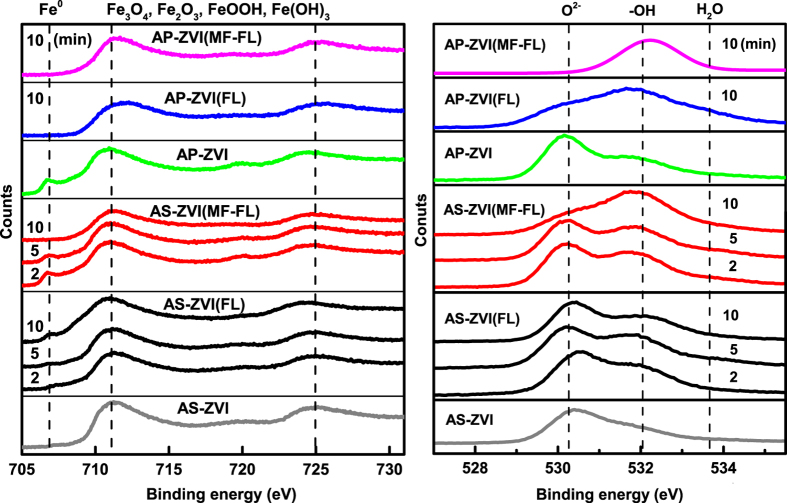
Time-dependent XPS spectra of the Fe 2p (left) and the O 1s (right) of the related ZVI samples (AS-ZVI and AP-ZVI), in the FL system and the MF-FL system, respectively.

Figure 7 exhibits the Fe 2p and O 1s XPS spectra of the related ZVI samples. The Fe^0^ peak at 706 eV of the pristine AS-ZVI was not detected, while the corresponding O 1s peak (O^2−^) appeared mainly at 530.2 eV. Considering the results of corresponding XRD characterization (Supplementary Fig. S10), it could be concluded that the AS-ZVI would have a structure of inner Fe^0^ wrapped by outer Fe_x_O_y_ layer^20^,^46^. After 2 min of reaction time, the MF-FL system instead of FL system leaded to an appearance of the Fe^0^ peak of the AS-ZVI particles. It suggested that MF could accelerate the destruction of oxides layer^18^, and exposure of inner Fe^0^. The decrease in the Fe^0^ peak intensity was also observed with reaction time elapsed from 2 to 10 min. It could ascribe to the precipitation of Fe^2+^ and Fe^3+^ on the reactive sites during the sample preparation procedure, since Fe^2+^ was still effectively released thereafter. The Fe^0^ peak of the AP-ZVI particles was of high intensity, indicating the serious breakdown of the oxides layer by intensive acid-pretreatment^1^. After 10 min, the Fe^0^ peak of the AP-ZVI particles disappeared in both the FL and MF-FL systems. It was because that the Fe^0^ reactive sites could be rapidly covered by *in-situ* generated iron oxides precipitates (Fig. 5e,f)^21^. The corresponding O 1s XPS spectra of the ZVI samples also evidenced that the surface dominant oxygen species would be conversed from oxides (O^2−^) to hydroxides (OH^−^) after 10 min reaction, except in the case of the FL system using AS-ZVI. It indicated that the pristine outer Fe_x_O_y_ layer would be relatively stable in the FL system without the introduction of MF.

### The proposed interactive role between MF and the pristine Fe_x_O_y_ layer

**Figure 8:**
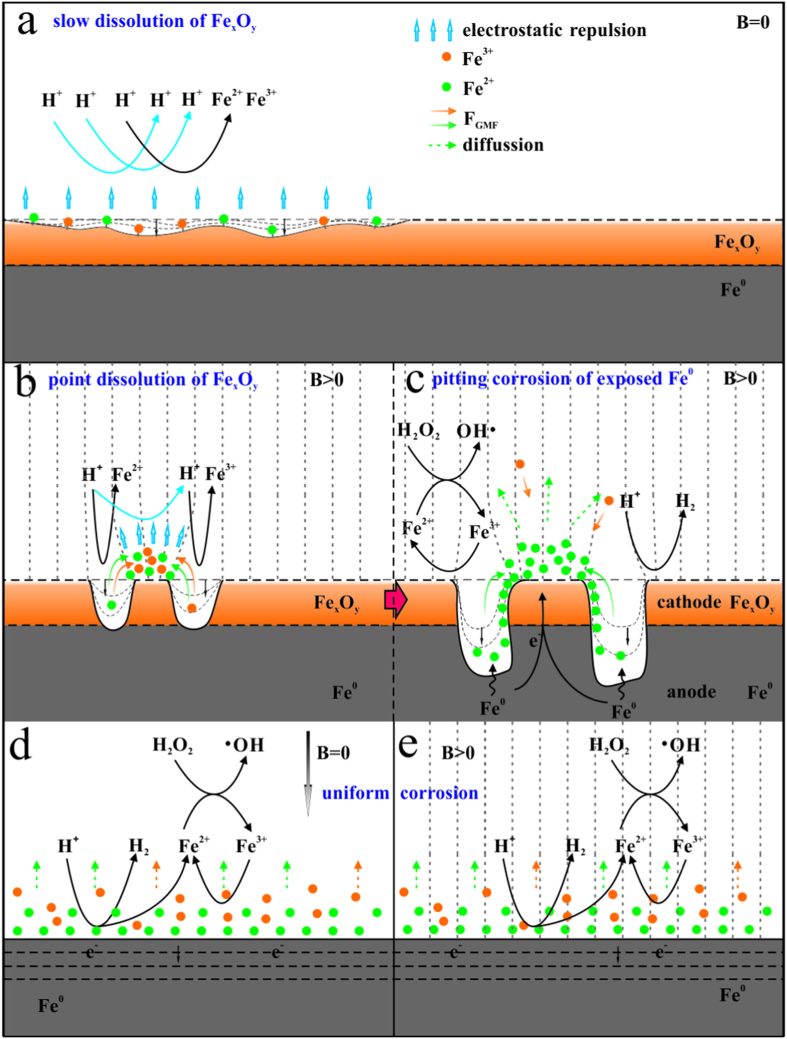
Schemes of the evolution mechanisms on the ZVI surface. (**a**) slow Fe_x_O_y_ dissolution (AS-ZVI, FL system); (**b**) initial rapid point dissolution of Fe_x_O_y_ followed by (**c**) pitting corrosion of the exposed Fe^0^ sites (AS-ZVI, MF-FL system); as well as similar uniform corrosion of AP-ZVI in (**d**) the FL system and (**e**) the MF-FL system.

Based on the above discussion, an interesting relationship between MF and the pristine Fe_x_O_y_ layer could be revealed as shown in Fig. 8. It was well established that the presence of the Fe_x_O_y_ layer could lead to strong inhibition of direct two-electron ZVI corrosion^20^. Therefore, the proton (H^+^) dissolution of the Fe_x_O_y_ layer would be the first step prior to further Fe^0^ corrosion (Fig. 8a)^1^,^47^. The dissolution procedure should be rather slow since the simultaneous production of surface-attaching Fe^2+^ and Fe^3+^ species would cause an electrostatic repulsion between H^+^ and the Fe_x_O_y_ surface^18^. Apparently, the Fe_x_O_y_-dependent heterogeneous Fenton-like reactions would be dominant in the FL system^32^, leading to rather long durations (>20 min) of the phase I (Fig. 1).

Figure 8b,c illustrate the proposed interactive role between the MF and the Fe_x_O_y_ layer in the MF-FL system. It comprised of two sequential procedures, i.e. a point dissolution step of the Fe_x_O_y_ surface followed by a pitting corrosion step of the unveiled Fe^0^ sites. Due to the ferromagnetic property of Fe^0^, the external MF could generate an inhomogeneous magnetic field on the particles surface^18^. Existence of the gradient magnetic force (GMF) would continuously drive the paramagnetic surface-attaching Fe^2+^ and Fe^3+^ to neighbor positions of higher field intensities^18^. As the migration of Fe^2+^ and Fe^3+^ proceeded, different surface sites with reallocated electrostatic repulsions would appear upon the Fe_x_O_y_ layer. The proton-dissolution would be more rapid on the sites of lower MF intensity, finally leading to the formation of two kinds of morphologic areas on the Fe_x_O_y_ surface, i.e. “disclosed” areas of fresh Fe^0^ and remained Fe_x_O_y_ areas attached with concentrated Fe^2+^/Fe^3+^ species (Fig. 8b).

Subsequently, MF-induced pitting corrosion of the “disclosed” Fe^0^ areas would occur (Fig. 8c), as the reactive Fe^0^ surface sites unveiled with the continuous point dissolution. Local action cells (i.e. Fe^2+^ concentration cells) would be generated due to the uneven distribution of Fe^2+^ in adjacent low-lying Fe^0^ and tuberculate Fe_x_O_y_ sites^18^,^48^. In the cell, the anode (the reactive Fe^0^ sites) would release Fe^2+^ on its surfaces and produce two free electrons simultaneously^48^. The electrons would be transferred to the surface of the counter Fe_x_O_y_ cathode where the reduction of H^+^ and Fe^3+^ happened^48^. Assisted by GMF, the *in-situ* generated Fe^2+^ could be continuously migrated along the particles surface from the anode to the cathode. It could thus maintain the electric potential difference of the Fe^2+^ concentration cells, causing efficient pitting corrosion of the reactive anodic Fe^0^ sites. In a summary, the Fe_x_O_y_-controlled heterogeneous Fenton-like reactions would be accelerated by the MF-induced point dissolution, leading to significantly shortened initial lag phase of 4-CP degradation in the MF-FL system. Then, excessive Fe^2+^ upon the cathodic tubercles would be diffused to the bulk solution during the pitting corrosion, accelerating the homogenous Fenton reaction and thereby enhancing the degradation of 4-CP.

Obviously, the Fe_x_O_y_ layer should play an important role in the MF-FL system. The interaction between the MF and the Fe_x_O_y_ layer was inevitable for the synergistic degradation of 4-CP. Acidic pretreatment could remove the Fe_x_O_y_ layer and expose abundant Fe^0^ sites, as well as promote the *in-situ* electron transfer from Fe^0^ to H^+^ or Fe^2+^. It would lead to a uniform and direct surface corrosion of the refreshed ZVI particles, as exhibited in Fig. 8d. However, the MF-caused local action cells would be rapidly eliminated due to the rapid electron transfer on the Fe^0^ surface, preventing the phenomenon of pitting corrosion^48^. The corrosion behaviors of AP-ZVI in the MF-FL (Fig. 8e) would be similar to that of the FL system, except that the GMF-directing accumulation of dissolved iron species would lead to iron precipitates at some specific sites (Fig. 5f).

## Conclusion

ZVI technologies is generally cost-effective, environmental friendly, and operation flexible. Unfortunately, formation of the Fe_x_O_y_ layers on the reactive surface of commercial ZVI materials should be unavoidable during their manufacture and storage. Use of MF in The loss of Fe^0^ surface reactivity will be a great challenge for the applications of ZVI technologies, either reduction or oxidation circumstances. This study demonstrated the synergistic 4-CP degradation achieved in a MF enhanced Fe^0^-catalyzed Fenton like system. It was found that the use of MF could effectively overcome the initial interfacial mass transport and significantly shorten the treatment duration. An amazing surface evolution mechanism on the pristine commercial ZVI particles was proposed, comprising of an initial MF-accelerated *in-situ* point dissolution of the Fe_x_O_y_ layer and a following pitting corrosion of the exposed Fe^0^ sites.

Application of MF in ZVI technologies will be attractive in practical wastewaters treatments, since the introduction of MF is commonly flexible and cost-effective. Under MF, commercial ZVI materials could be also directly acceptable. It would be unnecessary to inconvenient and costly pretreatment methods for removing unfavorable surface iron oxides. Nevertheless, the MF-leading enhancement on different ZVI decontamination processes adopting various types of ZVI materials e.g. natural iron-basing materials, are still uncertain. Therefore, the relationship between MF and the Fe_x_O_y_ characteristics (e.g. surface area and crystal types) is expected to be further revealed.

## Material and Methods

### Materials

All solutions were prepared in deionized water in this study. Commercial iron powders (≥98%) were obtained from Sinopharm Chemical Reagent Co., Ltd. (Shanghai, China) and stored in air about two years prior to use. Other chemicals, such as 4-cholophenol (4-CP, 99%), maleic acid (≥99%), p-benzoquinone (BQ, ≥98%), hydroquinone (HQ, ≥99%), catechol (CC, ≥98%), HCl, H_2_SO_4_, NaOH, methanol, acetonitrile, phosphoric acid, and formic acid were also purchased from Sinopharm Chemical Reagent. 4-chlororesorcinol (4-CR, 98%) and 4-chlorocatechol (4-CC, 97%) were supplied by Aladdin Chemistry Co., Ltd. and Sigma-Aldrich, respectively.

### Experimental setup and procedures

The experimental setup is illustrated in Supplementary Fig. S11. A borosilicate glass reactor (250 mL) was adopted with well mechanical-stirring and placed in a thermostatic water bath during the reaction. In the cases of the MF-FL system, two pieces of thin rounded rubber magnets (D = 20 mm, surface magnetized with field intensity ~60 mT) were assembled under the reactor to supply a magnetic field. The maximum magnetic field intensity in the reactor was 3.2 mT measured by a Gaussmeter (421-MNA-1904-VG, Lake Shore Cryotronics, Inc.). In a typical experiment, 200 mL solution containing predetermined concentration of 4-CP was prepared in the reactor with addition of certain amount of H_2_O_2_. The solution pH was adjusted by 0.05 M H_2_SO_4_ and 0.1 M NaOH prior to the reaction. Then the reaction was initiated by dosing ZVI powders. During the reaction, a mechanical stirrer (RW 20 digital, IKA®-Werke GmbH & CO. KG, Germany) was adopted to stir the solution and make most ZVI particles perform pseudo-circular motion near the bottom. At set intervals, samples were collected and filtered through 0.45 μm membrane immediately. Prior to analysis, a drop of methanol was added into the samples to stop the degradation reaction. For TOC analysis, a drop of 1.0 M NaOH was used instead. In certain cases, ZVI particles were collected after the reaction and rinsed by O_2_-free deionized water for several times to remove impurities on the ZVI surface. Afterwards, they were freeze-dried and stored in an anaerobic chamber before the surface characterizations. All experiments were conducted at least duplicates.

### Apparatus

4-CP and degradation intermediates were quantified by high performance liquid chromatography (HPLC, LC-15C, Shimadzu) equipped with a UV-Vis detector and a C18 column (WondaSil, 5 μm, 4.6 × 250 mm). Spin trapping examinations of hydroxyl radical were conducted with a Bruker EMXnano Electron spin resonance (ESR) spectrometer (Billerica, MA) at room temperature. The ESR spectrometer was operated under the conditions of MF 343 ± 10 mT, power 12.6 mW, modulation frequency 100 kHz, sweep time 30 s, and time constant 1.28 ms. Qualifications of degradation organic intermediates were conducted by high performance liquid chromatography-electrospray ionization-mass spectrometry (HPLC-ESI-MS, 1100, Agilent, USA) and gas chromatography-mass spectrometry (GC-MS, 7890A/5975C, Agilent). Their details were described in the Supplementary. Total organic carbon (TOC) and released chloride ions (Cl^−^) were measured by a TOC analyzer (multi N/C 2100, analytikjena, German) and an ion chromatography (ICS-1100, ThermoFisher), respectively. The concentration of ferrous and ferric ions was determined by the 1,10-phenanthroline colorimetric method with an UV-VIS spectrophotometer (UV-2600, Shimadzu) at maximum absorbance wavelength λ = 510 nm. In each analysis, 0.5 mL aqueous samples were filtered and immediately added into a 1 cm quartz cell containing 1 mL of 1,10-phenonthroline (2 g L^−1^), then diluted to a total volume of 3 mL by deionized water. As for the measurement of Fe(III) concentrations, hydroxylamine hydrochloride was adopted to pretreat the sample for reducing all Fe(III) into Fe(II) rapidly. Afterwards, the total Fe(II) concentration was measured by 1,10-phenanthroline colorimetric method. Then the Fe(III) concentration could be thus concluded as the subtraction concentration value of total dissolve iron to primary Fe(II) (i.e. [Fe(III)] = [total dissolved iron] − [Fe(II)].). The morphology of selected ZVI samples was characterized by a field emission scanning electron microscopy (FE-SEM, Nova NanoSEM 450, FEI) and an atomic force microscopy (AFM, SPM9700, Shimadzu, Japan). X-ray Photoelectron Spectra (XPS) were recorded by an X-ray photoelectron spectroscopy (Axis-Ultra DLD-600W, Shimadzu-Kratos) and X-ray powder diffraction (XRD) patterns were obtained on an X’Pert PRO diffractometer with Cu Kα radiation (λ = 1.5418 nm).

### Statistical analysis

All degradation experiments were carried out at triplicates, except the quantification experiments for the degradation intermediates and products that were conducted with duplicates. Statistical analyses were carried out using the software Origin 9.0 (©OriginLab Corporation). One-way ANOVA was used to analyze statistical significance of each treatment. Data were the means of replicates and error bars represented the standard deviation. P values of less than 0.05 were considered to be statistically significant. Correlation between the operational conditions has been assessed using the Pearson correlation coefficient (r) and P value for linear fitting. The results were presented by means ± standard error of the linear regression.

## Additional Information

**How to cite this article**: Xiang, W. *et al*. An insight in magnetic field enhanced zero-valent iron/H_2_O_2_ Fenton-like systems: Critical role and evolution of the pristine iron oxides layer. *Sci. Rep.* **6**, 24094; doi: 10.1038/srep24094 (2016).

## Supplementary Material

Supplementary Information
