# Supplementary material for: An insight in magnetic field enhanced zero-valent iron/H2O2 Fenton-like systems: Critical role and evolution of the pristine iron oxides layer
Source: Sci Rep. 2016 Apr 7;6:24094. doi: 10.1038/srep24094 (PMC4823663; doi:10.1038/srep24094)
Supplement: Supplementary Information [file srep24094-s1.pdf]

Supplementary information

An insight in magnetic field enhanced zero-valent iron/H_2O_2 Fenton-like systems: Critical role and evolution of the pristine iron oxides layer

Wei Xiang[1], Beiping Zhang[1,*], Tao Zhou[1,*], Xiaohui Wu[1], Juan Mao[1]

A. Supplementary Tables

Table S1 One-way ANOVA analyses on 4-CP removal data (different parameters).

Table S2 One-way ANOVA analyses on 4-CP removal data (for MF effect).

Table S3 The statistical test of E_a values from the FL and MF-FL systems.

B. Supplementary Figures

Figure S1 Comparative 4-CP degradation in the FL and MF-FL systems at different ZVI dosages.

Figure S2 Comparative 4-CP degradation in the FL and MF-FL systems at different H_2O_2 dosages.

Figure S3 Comparative 4-CP degradation in the FL and MF-FL systems at different initial pH.

Figure S4 Comparative 4-CP degradation in the FL and MF-FL systems under different temperature.

Figure S5 An example of the two-phase identification of 4-CP degradation.

Figure S6 Time-dependent ESR spectra obtained from (a) FL and (b) MF-FL systems, and (c) the related normalized evolution patterns of hydroxyl radical (For each sample, 50 mM DMPO was added as the trapping reagent).

Figure S7 Examinations of (a) maleic acid, (b) benzoquinone and (c) hydroquinone by HPLC.

Figure S8 The simultaneous evolutions of dissolved iron species and pH in the FL and MF-FL systems based on AS-ZVI and AP-ZVI, respectively.

Figure S9 AFM images (2D) of related ZVI samples and the relevant surface height distributions.

Figure S10 XRD patterns of pristine ZVI, acid pretreated ZVI, and related ZVI samples collected after reaction.

Figure S11 Experimental setup of the MF-FL system for the 4-CP degradation.

C. Supplementary methods

Analytic methods for 4-CP's intermediates and products.

Preparing procedure of the acid-pretreated ZVI particles.

Table S1. One-way ANOVA analyses for the 4-CP removal data (different parameters).

Parameters	No. of groups	System		F	P	Effect *
ZVI dosage	4 (0.025, 0.05, 0.1 and 0.5 mg/L)	FL	Phase I	4.99	0.057	I
			Phase II	10.53	0.013	S
		MF-FL	Phase I	27.94	0.004	S
			Phase II	248.58	0.000	S
H_2O_2 dosage	5 (0.5, 1, 2, 3.5 and 5 mM)	FL	Phase I	16.60	0.009	S
			Phase II	7.33	0.025	S
		MF-FL	Phase I	1385.83	0.000	S
			Phase II	549.98	0.000	S
Initial pH	4 (2, 3, 4 and 5)	FL	Phase I	57.74	0.001	S
			Phase II	3.18	0.147	I
		MF-FL	Phase I	145.28	0.000	S
			Phase II	271.61	0.000	S
Temperature	4 (10, 20, 30 and 40 ℃)	FL	Phase I	163.71	0.000	S
			Phase II	33.19	0.003	S
		MF-FL	Phase I	7.83	0.039	S
			Phase II	55.25	0.001	S

* S: Statistically significant at the level of 0.05; I: Statistically insignificant at the level of 0.05.

Table S2. One-way ANOVA analyses for the 4-CP removal data (MF effect).

Target	No. of groups	Parameters	Level	No. of phases	F	P	Effect *
Magnetic field	2 (FL and MF-FL systems)	ZVI dosage (mg/L)	0.025	Phase I	2.39	0.262	I
				Phase II	83205	0.000	S
			0.05	Phase I	520.2	0.002	S
				Phase II	52.79	0.018	S
			0.1	Phase I	21.07	0.044	S
				Phase II	212.21	0.005	S
			0.5	Phase I	74.93	0.003	S
				Phase II	179.63	0.001	S
		H_2O_2 dosage (mM/L)	0.5	Phase I	16.2	0.056	I
				Phase II	13.71	0.066	I
			1	Phase I	520.20	0.002	S
				Phase II	52.78	0.018	S
			2	Phase I	592.99	0.002	S
				Phase II	1043.84	0.001	S
			3.5	Phase I	-	-	-
				Phase II	21.20	0.044	S
			5	Phase I	-	-	-
				Phase II	-	-	-
		Initial pH	2	Phase I	87.46	0.011	S
				Phase II	98.50	0.010	S
			3	Phase I	520.20	0.002	S
				Phase II	52.78	0.018	S
			4	Phase I	4.55	0.167	I
				Phase II	0.82	0.460	I
			5	Phase I	-	-	-
				Phase II	-	-	-
		Temperature (℃)	10	Phase I	4	0.184	I
				Phase II	41.50	0.023	S
			20	Phase I	520.20	0.002	S
				Phase II	52.78	0.018	S
			30	Phase I	3.39	0.207	I
				Phase II	25.83	0.037	S
			40	Phase I	11.81	0.075	I
				Phase II	222.20	0.004	S

* S: Statistically significant at the level of 0.05; I: Statistically insignificant at the level of 0.05.

-: Not applicable.

Table S3 The statistical test of E_a values from the FL and MF-FL systems.

	DF	Sum of Squares	Mean Square	F Value	P Value
**Factor A** *	1	186.3225	186.3225	102.2346	0.06276
**Factor B** *	1	2782.563	2782.563	1526.783	0.01629
**Model**	2	2968.885	1484.443	814.5089	0.02477
**Error**	1	1.8225	1.8225	--	--
**Corrected Total**	3	2970.708	--	--	--

* Factor A: In the absence or presence of MF; Factor B: During the phase I or II.

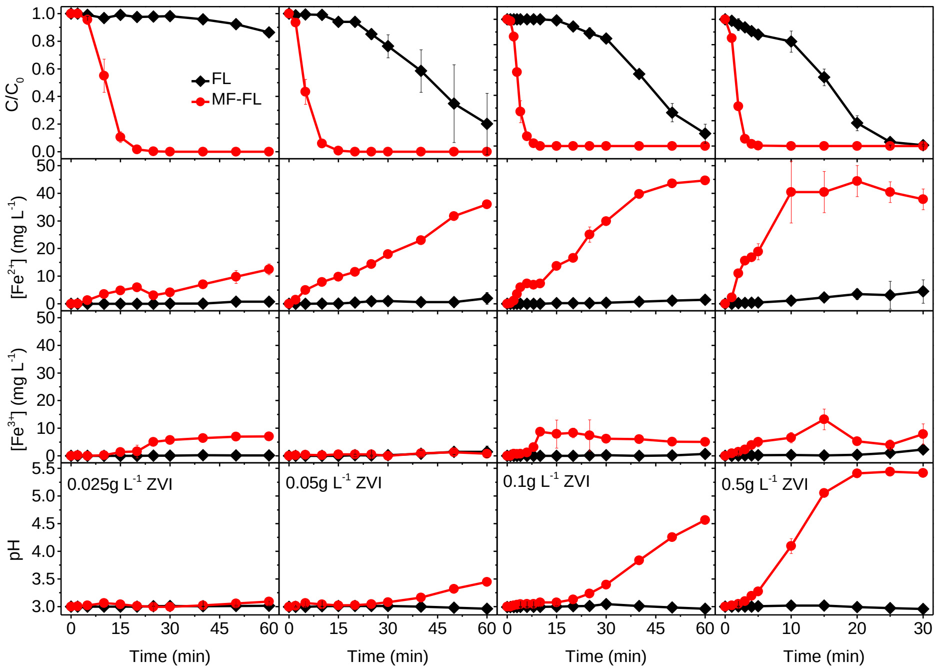

Figure S1. Comparative 4-CP Degradation in the FL and MF-FL systems at different ZVI dosages.(Conditions: pH_0 3, 20 ℃, H_2O_2 1.0 mM, 4-CP 25 mg L^{-1}, ZVI 0.025, 0.05, 0.1 and 0.5 g L^{-1}.) The error bars represent the standard deviation based on triplicate experiments.

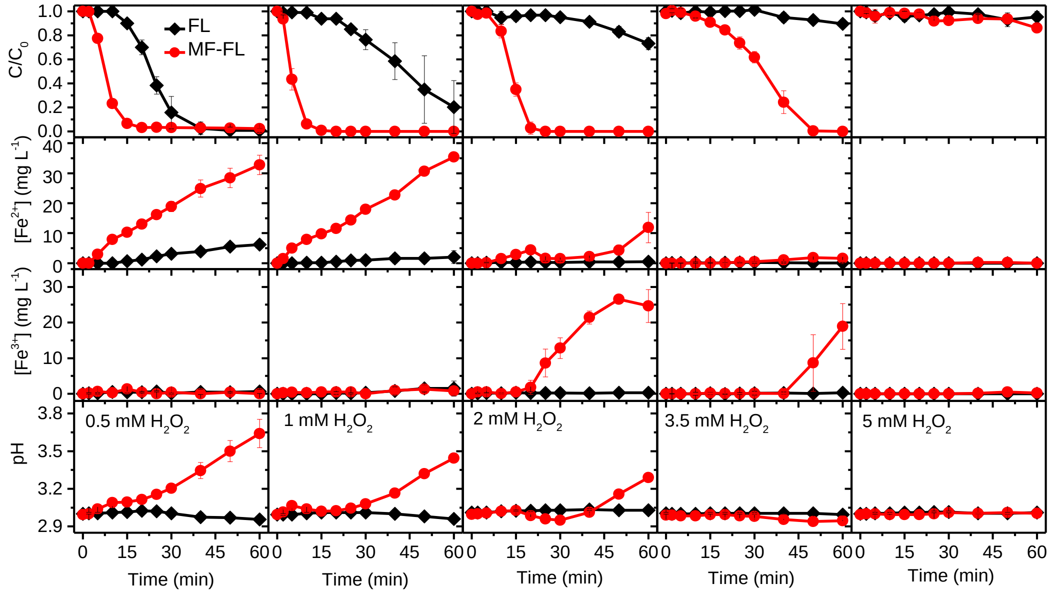

Figure S2. Comparative 4-CP Degradation in the FL and MF-FL systems at different H_2O_2 dosages. (Conditions: pH_0 3, 20 ℃, ZVI 0.05 g L^{-1}, 4-CP 25 mg L^{-1}, H_2O_2 0.5, 1, 2, 3.5 and 5mM.) The error bars represent the standard deviation based on triplicate experiments.

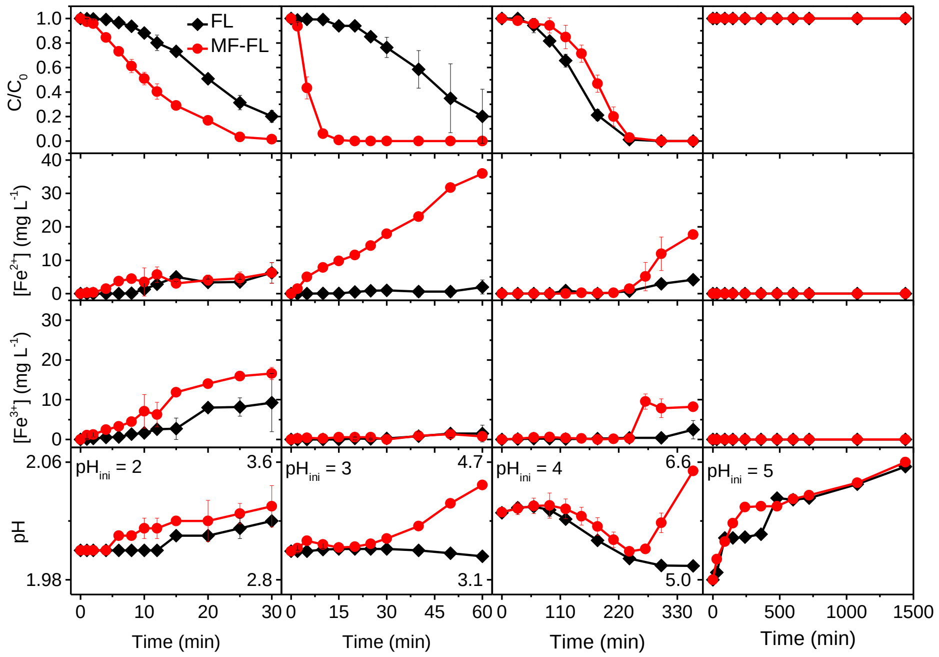

Figure S3. Comparative 4-CP Degradation in the FL and MF-FL systems at different initial pH.(Conditions were: 20 ℃, 0.05 g L^{-1} ZVI, 4-CP 25 mg L^{-1}, H_2O_2 1.0 mM, pH_0 2, 3, 4 and 5.) The error bars represent the standard deviation based on triplicate experiments.

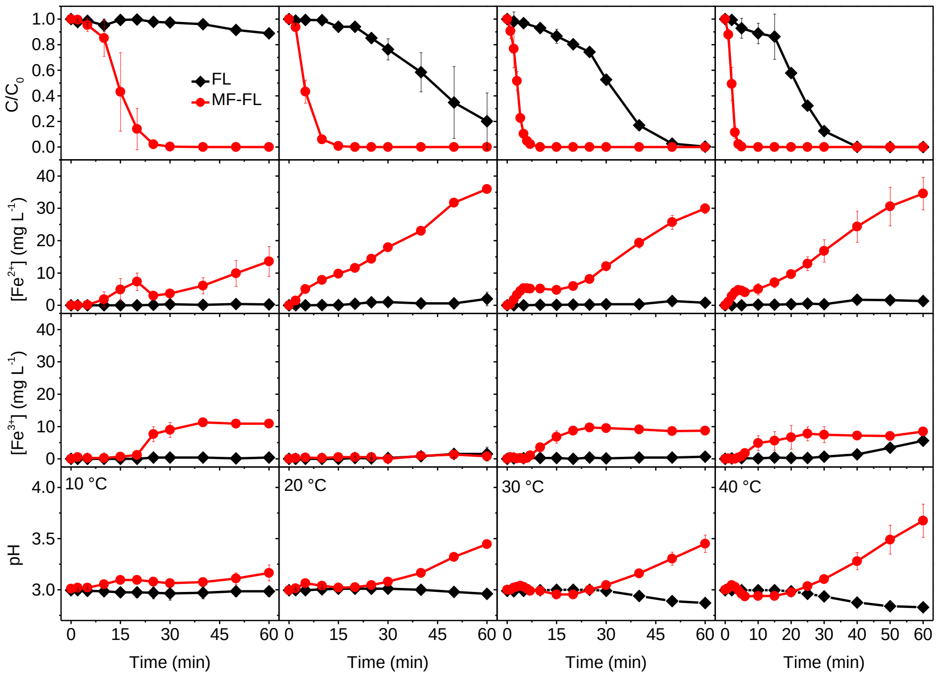

Figure S4. Comparative 4-CP Degradation in the FL and MF-FL systems under different temperature. (Conditions were: pH_0 3, ZVI 0.05 g L^{-1}, 4-CP 25 mg L^{-1} and H_2O_2 1.0 mM, reaction temperature 10, 20, 30 and 40 ℃.) The error bars represent the standard deviation based on triplicate experiments.

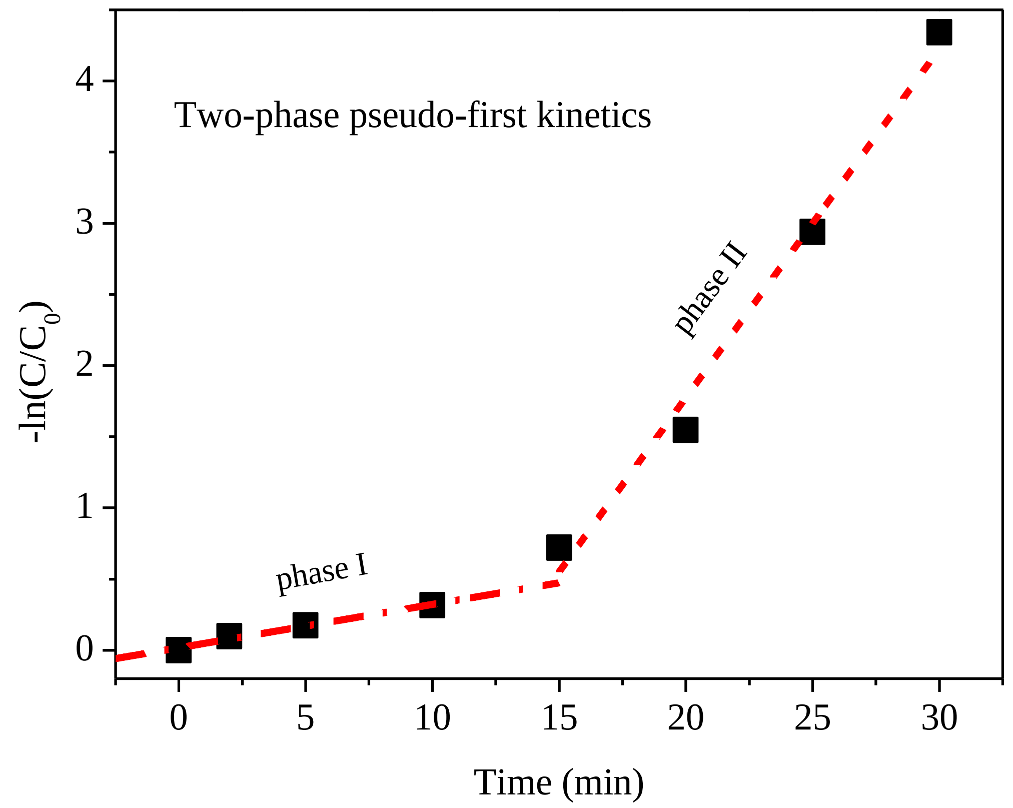

Figure S5. An example of the two-phase identification of 4-CP degradation. (FL system, with 0.5 g L^{-1} ZVI, 20 ℃, 1 mM H_2O_2 and 25 mg L^{-1} 4-CP.)

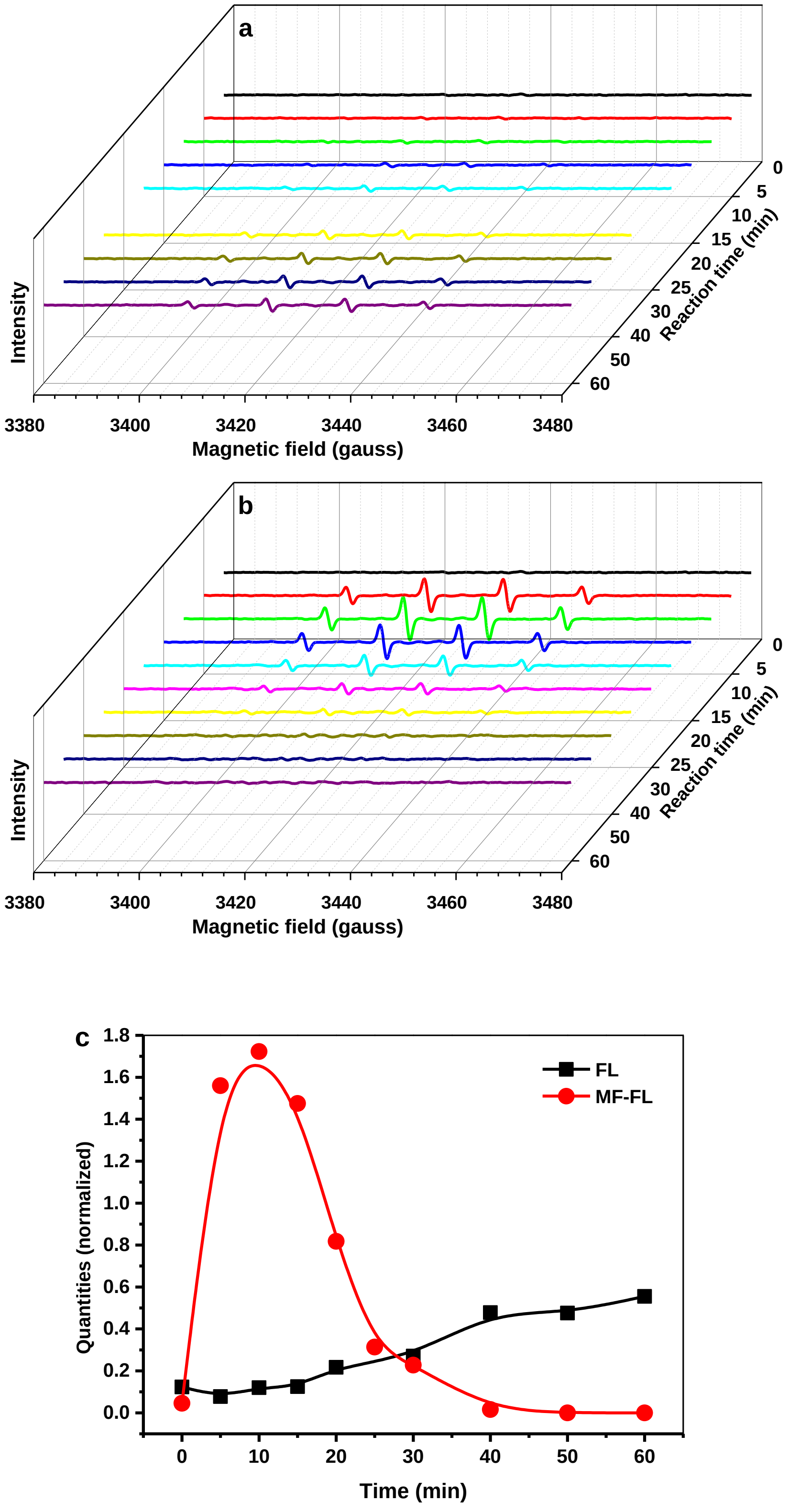

Figure S6. Time-dependent ESR spectra obtained from (a) FL and (b) MF-FL systems, and (c) the related normalized evolution patterns of hydroxyl radical (For each sample, 50 mM DMPO was added as the trapping reagent).

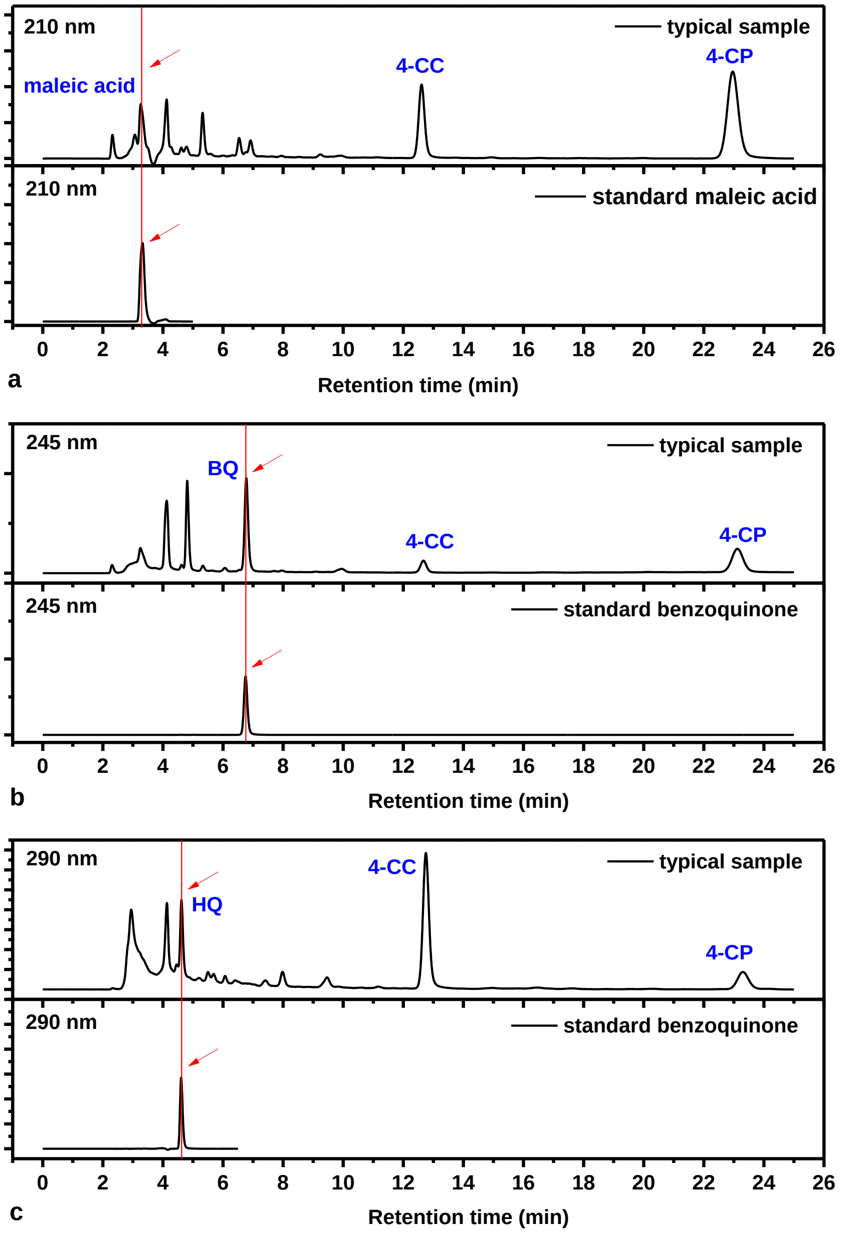

Figure S7. Examinations of (a) maleic acid, (b) benzoquinone and (c) hydroquinone by HPLC.

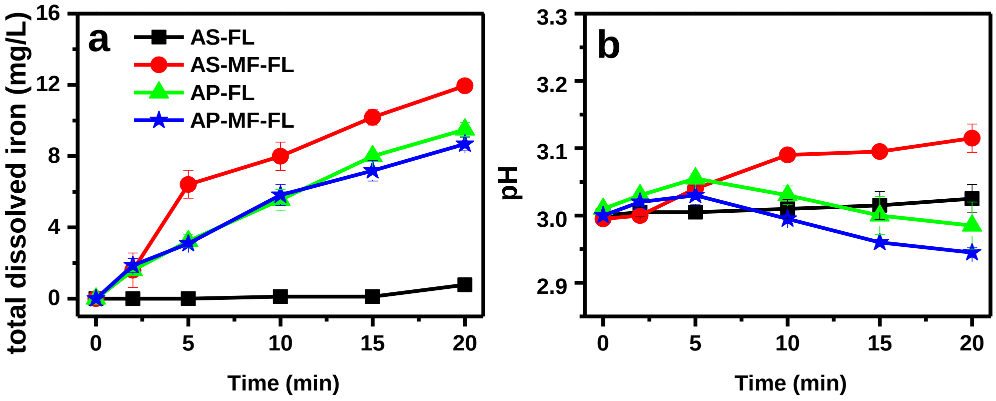

Figure S8. The simultaneous evolutions of (a) dissolved iron species (b) pH, corresponding to the FL and MF-FL systems based on AS-ZVI and AP-ZVI, respectively. The error bars represent the standard deviation based on triplicate experiments.

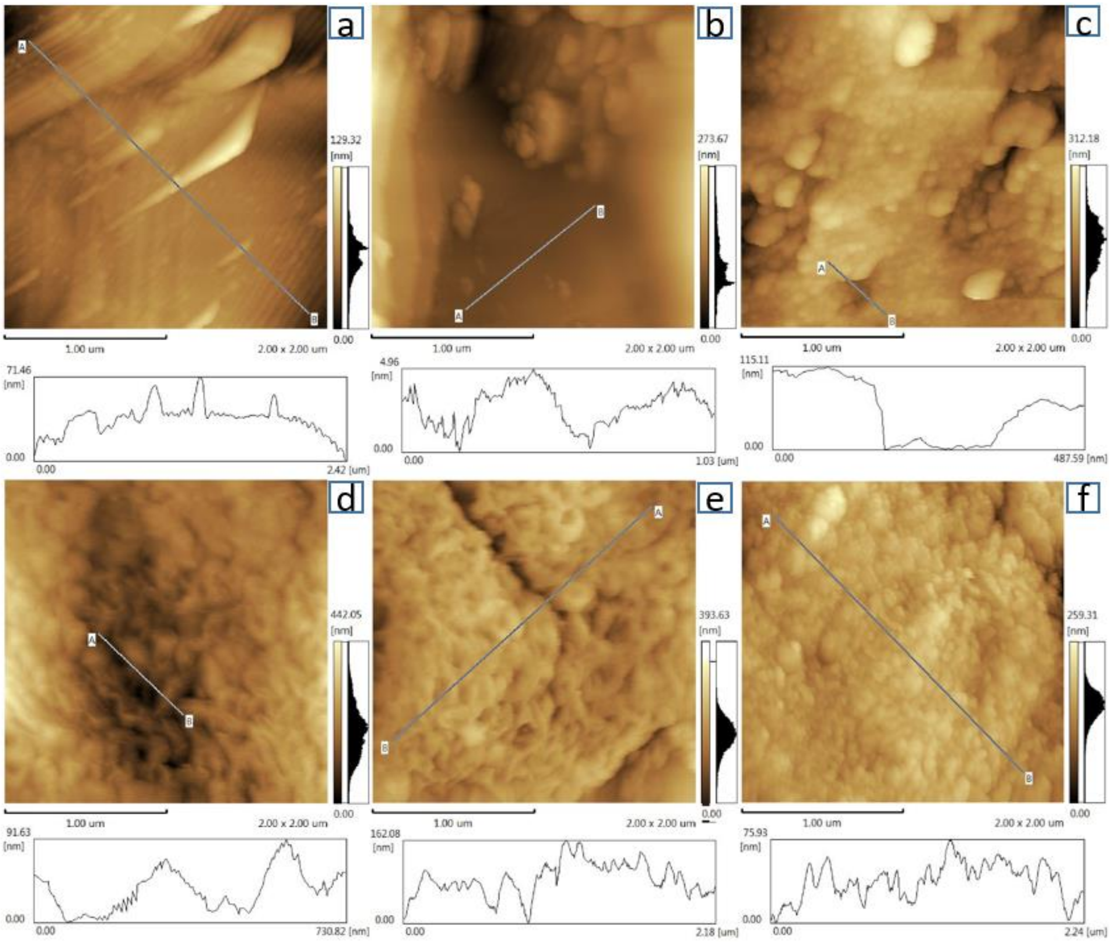

Figure S9. AFM 2D images of(a) the pristine AS-ZVI (b) the reacted AS-ZVI (FL, 10 min) (c) the reacted AS-ZVI (MF-FL, 10 min), (d) the original AP-ZVI, (e) the reacted AP-ZVI (FL, 10 min),and (f) the reacted AP-ZVI (MF-FL, 10 min).The relevant surface height distributions along the lines (A-B) plotted in the images are also given.

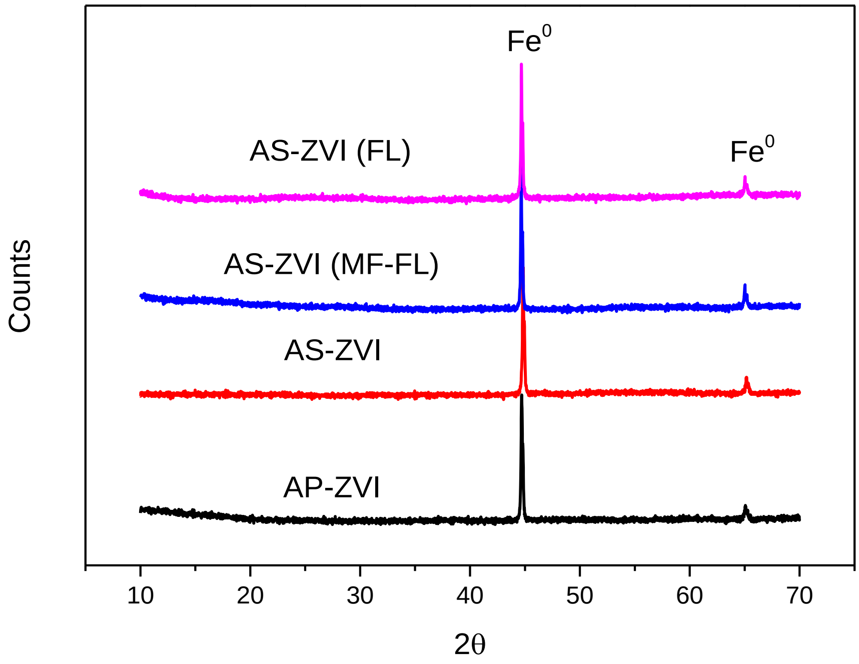

Figure S10. XRD patterns of the related ZVI samples.

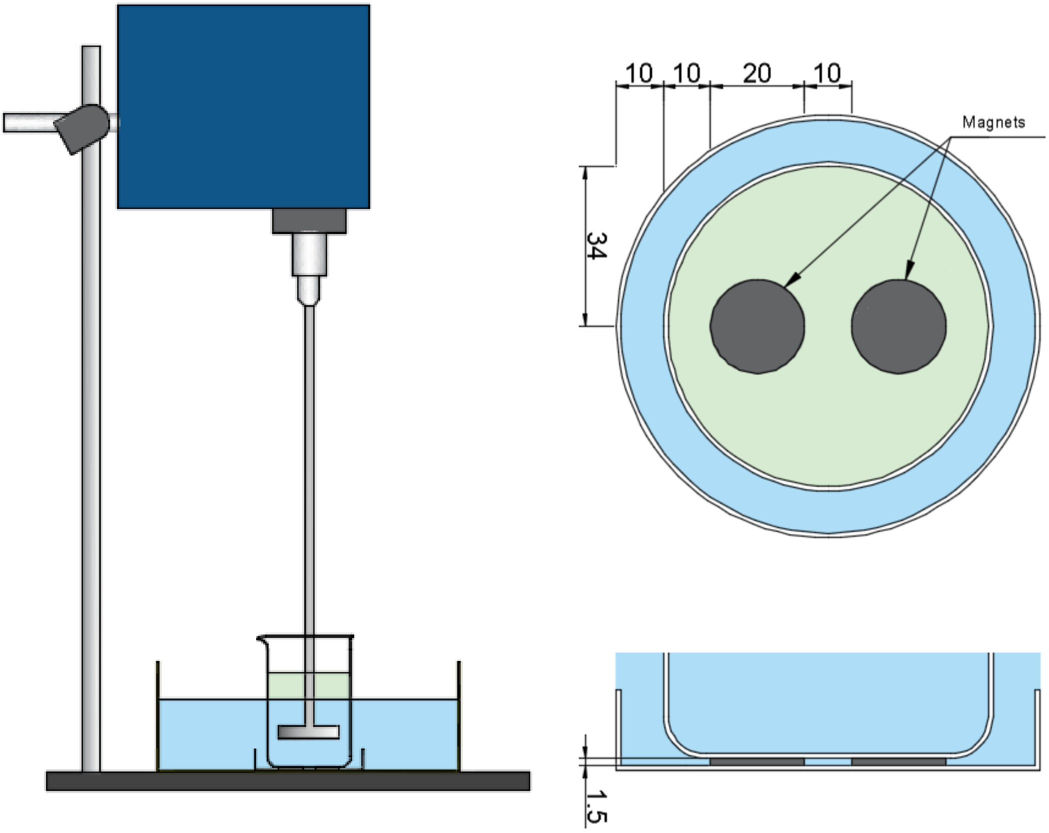

Figure S11. Experimental setup of the MF-FL system for the 4-CP degradation.

Supplementary methods

Analytic methods of 4-CP's intermediates and products

To detect the intermediates of 4-CP degradation, the initial 4-CPconcentration was increased to 50 mg L^{-1}. The intermediates were qualitatively identified by a gas chromatograph-mass spectrometry (GC-MS, 5975C, Agilent, USA) and a liquid chromatography-electrospray ionization tandem-mass spectrometry (LC-ESI-MS, 1100 LC/MSD Trap, Agilent, USA). A high performance liquid chromatography (HPLC) was used to quantitatively examine the intermediates of 4-CP using the external standards of six purchased pure intermediates.

For GC-MS analysis, an Agilent DB-17 capillary column (30 m × 0.25 mm × 0.50 μm) was utilized. The temperature of the injection block was 300 ℃; the oven temperature program was 60 ℃, rinsing at 10 ℃ min^{-1} to a final temperature of 280 ℃. Helium was used as the carrier gas at the flow rate of 1 $mLmin^{-1}$.

For LC-ESI-MS analysis, a C18 column (WondaSil, 5μm, 4.6 × 250 mm) was used with a mixture mobile phase of acetonitrile (30%) and 0.1% formic acid solution (70%), and the flow rate was 0.9 mL min^{-1}. ESI-MS analysis was carried out with the ion-transfer capillary temperature of 300 ℃, and the spray voltage was 45 kV.

For HPLC analysis, the C18 column (WondaSil, 5μm, 4.6 ×250 mm) was used with a mixture mobile phase of acetonitrile (30%) and 0.1% phosphoric acid solution (70%). Samples were analyzed at a flow rate of 0.9 mL min^{-1} and UV absorbance wavelength of 210, 245, 267 and 290 nm, respectively for maleic acid, BQ, 4-CR and HQ, as well as 280 nm for 4-CP, CC and 4-CC.

Preparation of acid-pretreated ZVI

The acid-pretreated ZVI particles was prepared as follows. 2 g of commercial ZVI powders was added into a 100mL serum containing 60 mL of 1 M HCl. Then the solution was mechanically stirred for 5min. The collected ZVI particles were wash repeatedly by O_2-free deionized water until the suspension mixtures reached neutral. Afterwards, the separated ZVI particles were freeze-dried overnight and reserved in the anaerobic chamber prior to use.
